# Long-term quality of life and depressive symptoms among survivors of poor-grade aneurysmal subarachnoid hemorrhage

**DOI:** 10.1016/j.bas.2026.107465

**Published:** 2026-10-09

**Authors:** Isabel David, Ibrahim E. Efe, Igor Fischer, David Wasilewski, Sihmehmet Sahan, Yousef Abusabha, Milad Neyazi, Rajiv Kumar Khajuria, Katharina Faust, Sajjad Muhammad

**Affiliations:** Department of Neurosurgery, Düsseldorf University Hospital, Heinrich-Heine University Düsseldorf, Düsseldorf, North Rhine-Westphalia, 40225, Germany

**Keywords:** Aneurysmal subarachnoid hemorrhage, Poor-grade subarachnoid hemorrhage, Quality of life, Functional recovery, Depressive symptoms, Long-term outcomes

## Abstract

**Introduction:**

Poor-grade aneurysmal subarachnoid hemorrhage (aSAH) is associated with high mortality and poor functional outcomes. Despite improved survival with advances in neurocritical care, long-term quality of life (QOL) and psychological sequelae remain insufficiently characterized.

**Research question:**

What is the long-term quality of life among survivors of poor-grade aSAH and how is it associated with functional and psychological outcomes?

**Material and methods:**

We retrospectively analyzed patients with poor-grade aSAH (WFNS IV–V) treated between 2013 and 2023. Survivors underwent long-term follow-up assessment using the Stroke-Specific Quality of Life scale (SS-QOL), SF-12 Physical Component Summary (PCS), and Patient Health Questionnaire (PHQ-9). SS-QOL >147 was used as descriptive threshold.

**Results:**

Of 339 patients with poor-grade aSAH, 47.8% died. Among survivors, 15.9% were lost to follow-up. Among eligible survivors, 35.8% did not participate, resulting in 79 included patients (23.3% of the original cohort; median follow-up of 4.36 years. Among participants, 88.6% had SS-QOL >147. SS-QOL correlated positively with SF-12 PCS (ρ = 0.49, p < 0.001) and negatively with PHQ-9 (ρ = −0.67, p < 0.001). In multivariable analysis, PHQ-9 was the only variable associated with SS-QOL. Higher depressive symptom burden was associated with lower scores across all SS-QOL domains.

**Discussion and conclusion:**

Among selected survivors of poor-grade aSAH, favorable QOL was observed despite severe initial disease. Depressive symptom burden was strongly associated with overall QOL, and inversely associated with all SS-QOL domains. However, mortality, loss to follow-up, and non-participation represent sources of selection bias and may result in overestimation of QOL.

## Highlights

• Many poor-grade SAH survivors achieved favorable long-term quality of life.
• Lower QOL was most strongly reflected in difficulties with independence and daily functioning.
• Functional recovery was closely linked to patients' perceived quality of life.
• Physical health was positively associated with quality of life.
• Depressive symptoms were strongly associated with poorer quality of life.

## 1 Introduction

Aneurysmal subarachnoid hemorrhage (aSAH) is associated with high mortality and often devastating neurological outcomes, particularly in patients presenting with poor clinical grade (WFNS IV-V). Despite advances in neurocritical care and aneurysm treatment, which have improved survival, a substantial proportion of patients experience long-term disability (Rinkel and Algra, 2011; Hammer et al., 2018).

Outcome assessment in poor-grade aSAH has traditionally focused on clinician-reported functional measures such as the modified Rankin Scale (mRS), Barthel Index, or Glasgow Outcome Scale (GOS) (Schatlo et al., 2021; Zhao et al., 2017; Schwartz et al., 2017). Several studies have demonstrated that a subset of patients can achieve functional independence despite an initially poor clinical presentation. However, these metrics provide limited insight into patients’ subjective well-being and may fail to capture important aspects of quality of life and psychological burden (Tang et al., 2020; Passier et al., 2011).

Previous studies have demonstrated that survivors of aSAH frequently experience reduced QOL and depressive symptoms, with approximately one-third of patients reporting impaired QOL at one year after hemorrhage (Taufique et al., 2016). However, reported outcomes vary substantially, as other studies indicate that up to 85% of poor-grade survivors achieve favorable quality of life (Schwartz et al., 2017; Goldberg et al., 2023). These wide variations likely reflect differences in patient selection, outcome instruments, definitions of favorable QOL, and follow-up duration, limiting direct comparability across studies. Despite these findings, the factors shaping long-term patient-reported outcomes in this population remain incompletely understood. Interpretation of these studies is further complicated by substantial mortality and survivorship bias, as patient-reported outcomes are necessarily restricted to long-term survivors.

To address these limitations, validated patient-reported outcome measures are required. The Stroke-Specific Quality of Life scale (SS-QOL) enables comprehensive assessment across functional and psychosocial domains, while the Patient Health Questionnaire-9 (PHQ-9) provides a standardized evaluation of depressive symptoms.

The aim of this study was to assess long-term QOL and depressive symptoms in survivors of poor-grade aSAH and to identify factors associated with reduced QOL. We hypothesized that lower SS-QOL scores would be associated with reduced physical function and higher depressive symptom burden among long-term survivors of poor-grade aSAH.

This study addresses an important gap in the existing literature by characterizing long-term patient-reported outcomes in a strictly defined cohort of poor-grade aSAH survivors using both domain-specific SS-QOL assessment and standardized evaluation of depressive symptoms. In addition to overall QOL, we examined individual SS-QOL domains and their clinical correlates to identify specific dimensions of long-term health-related QOL that may be particularly affected in this population.

## 2 Methods

### 2.1 Study design

This was a retrospective, single-center cohort study with cross-sectional follow-up assessment. Clinical and radiological data were extracted from patient records; QOL and depression were evaluated at follow-up using standardized questionnaires. The study was approved by the Ethics Committee of the medical faculty at Heinrich-Heine University Düsseldorf (Ethics approval number: 2024–2822). All participants gave written informed consent.

### 2.2 Study population

Patients with aSAH admitted to the Department of Neurosurgery, University Hospital Düsseldorf, between January 2013 and December 2023 were included. Inclusion criteria: age ≥ 18 years, poor-grade aSAH (WFNS IV or V), onset of hemorrhage within the study period, and treatment at our institution. Exclusion criteria: traumatic subarachnoid hemorrhage, incomplete records, death during initial hospitalization, inability to participate due to severe functional impairment, or missing follow-up data. Patients requiring institutional care were not formally excluded by protocol; however, most were unable to participate due to severe cognitive or physical limitations that prevented informed consent or completion of the questionnaire. Eligible survivors were identified from institutional records and contacted by telephone and/or mail. Patients who agreed to participate provided written informed consent before completing the study questionnaires. In total, 79 patients fulfilled the criteria and agreed to participate in this study (Fig. 1).

**Fig. 1:**
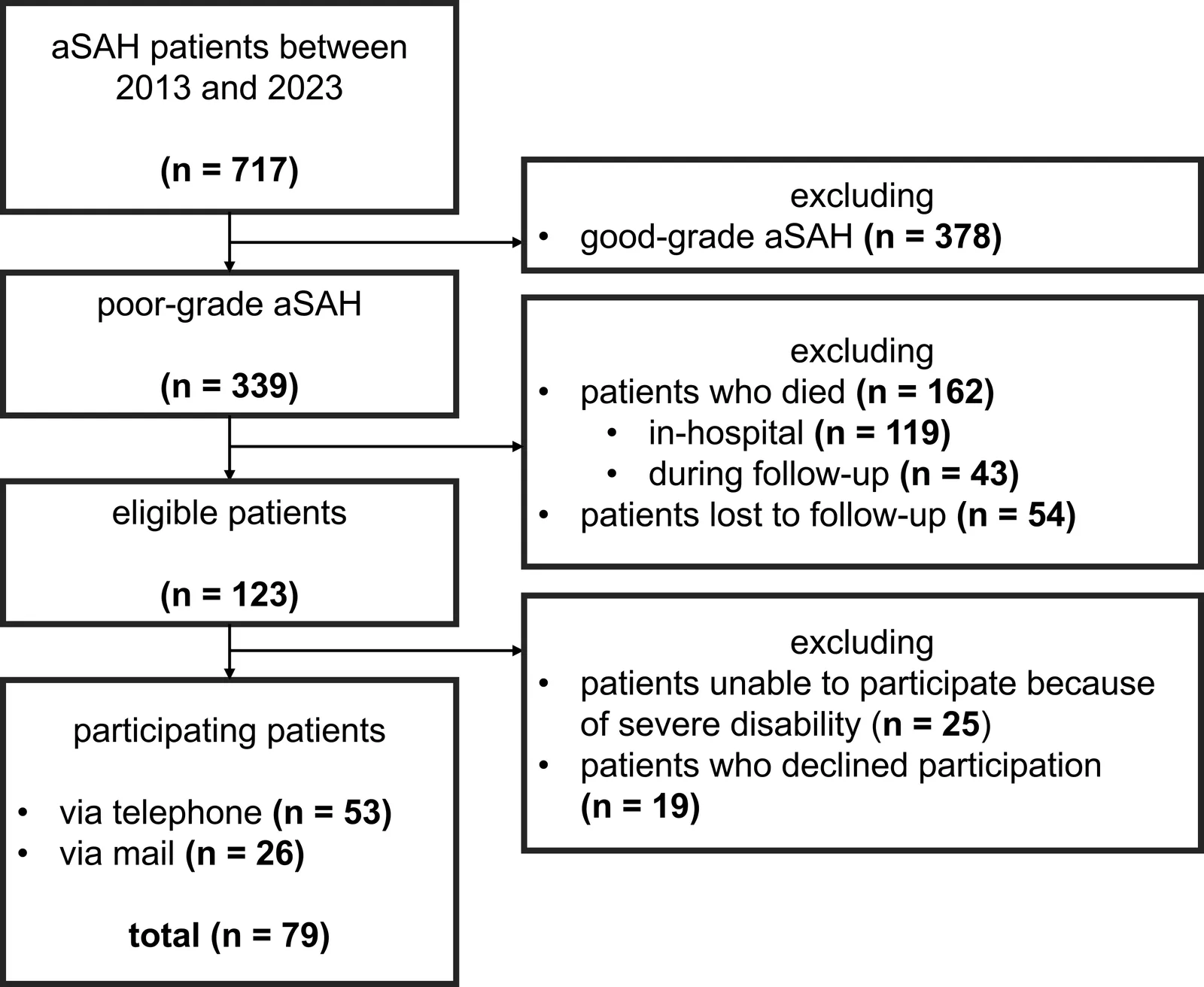
Flowchart detailing patient selection.

### 2.3 In-hospital management

All patients were managed under a standardized institutional pathway aligned with international guidelines. Hemorrhage severity was graded using the modified Fisher scale. Surgical clipping or endovascular intervention was performed within 24 h or subsequently after interdisciplinary evaluation. Emergency clipping with hematoma evacuation was performed when mass effect mandated decompression. Patients with GCS ≥13 were monitored with a low-stimulation strategy until aneurysm occlusion; GCS ≤12 or radiographic intracranial hypertension prompted anesthesia, intubation, and external ventricular drainage. Post-treatment protocol included cranial CT with CT-angiography (CTA) and CT-perfusion (CTP) at 6 h, then serial CTP on days 4, 7, and 10–11, or sooner if cerebral vasospasm (CVS) or delayed cerebral ischemia (DCI) was suspected. CVS and DCI were managed with induced hypertension and selective intra-arterial therapy when appropriate. Intravenous (IV) nimodipine treatment was standard. The patients who did not tolerate IV nimodipine were treated with standard oral dose of nimodipine. Functional outcome was assessed using the Glasgow Outcome Scale (GOS) at hospital discharge. GOS was not reassessed during long-term follow-up.

### 2.4 Quality of life assessment

QOL was assessed using SS-QOL and the physical component of SF-12. Depression was assessed with PHQ-9. For telephone interviews, study personnel read all SS-QOL, SF-12, and PHQ-9 items verbatim and recorded the patients' responses. For mailed questionnaires, patients completed the forms independently. Assistance from relatives was permitted only for reading or writing support and not for interpreting or answering the questions. All responses reflected the patients’ own answers. Patient-reported outcomes were assessed at least 12 months after hospital discharge. Median follow-up was 4.36 years.

**Stroke-Specific Quality of Life (SS-QOL):** A self-report instrument with 49 questions across 12 domains (energy, family roles, language, mobility, mood, personality, self-care, social roles, thinking, upper extremity function, vision, work/productivity). Each item is rated on a five-point Likert scale (higher = better QOL). Total score range 49–245; ≤147 indicates reduced QOL (Williams et al., 1999).

**Short Form-12 Health Survey (SF-12):** The Physical Component Summary (PCS) was used. Scoring followed standardized guidelines (Ware et al., 1996; Lin et al., 2020).

**Patient Health Questionnaire (PHQ-9):** Nine items (0–3 each) corresponding to DSM-5 depression criteria. Total score ≥10 indicates at least moderate depressive symptomatology (Löwe et al., 2004; Kroenke et al., 2001).

### 2.5 Statistical analysis

Data from 79 patients were analyzed in Python, using statsmodels and Pingouin statistical libraries. Variables were summarized as frequencies (n) and percentages (%). For descriptive analysis, patients were stratified according to an SS-QOL threshold of 147 points, corresponding to 60% of the maximum attainable score, as previously reported in the literature, resulting in two groups: SS-QOL >147 vs. SS-QOL ≤147 (Williams et al., 1999; Ramos-Lima et al., 2018). Statistical significance was defined as p < 0.05. Spearman's rank correlation was used to assess associations between SS-QOL and SF-12 PCS and SS-QOL and PHQ-9, with 95% confidence intervals reported. Multiple linear regression was used to assess associations between SS-QOL and clinical variables, including sex, age, WFNS score, mRS score at discharge, length of follow-up, history of depression, and total PHQ-9 score. The associations between the PHQ-9 score and individual SS-QOL domain scores were assessed using linear regression and Spearman's rank correlation. For comparability, all SS-QOL domain scores were normalized to the range [0, 1]. Regression residuals were assessed for normality using D'Agostino-Pearson test and for homoscedasticity using Breusch-Pagan test. The Benjamini-Hochberg false discovery rate (FDR) was used to correct for multiple testing.

## 3 Results

### 3.1 Cohort and baseline characteristics

A total of 717 aSAH patients were screened, of whom 339 (47.3%) had poor-grade aSAH (WFNS IV–V). Based on hospital records, 162 poor-grade patients (47.8%) died either during initial hospitalization or follow-up. Causes of death included early brain injury (n = 48, 29.6%), DCI (n = 71, 43.8%), aSAH-related complications beyond 28 days (n = 27, 16.7%), and non-aSAH-related mortality (n = 16, 9.9%).

Fifty-four patients (15.9%) were lost to follow-up, leaving 123 eligible survivors (36.3%). Of these, 44 (35.8%) did not participate due to severe functional impairment requiring institutional care (n = 25) or personal reasons (n = 19). The final study cohort comprised 79 patients (23.3% of the original poor-grade cohort) (Fig. 1).

Among the included patients, 61 (77%) were female, 18 (23%) were male, and the mean age was 52 ± 11 years. WFNS grade IV was present in 39 (49%), and grade V in 40 (51%) patients. Aneurysm location was most commonly the anterior communicating artery (32%), followed by the middle cerebral artery (24%), posterior communicating artery (12%), posterior inferior cerebellar artery (9%), the internal carotid artery (9%), and others (14%). Surgical clipping was performed in 51 (65%) patients, whereas endovascular coiling or other treatment strategies were performed in 25 (31%) and 3 (4%) patients, respectively. The modified Fisher grade was 4 in 53 (67%) patients, grade 3 in 22 (28%) patients, and grade 2 in 4 (5%) patients. On the Glasgow Outcome Scale (GOS), 27 (34%) patients had a favorable outcome (GOS 4-5) at the time of discharge. The median mRS score at discharge was 4 (IQR, 2 – 4) (Table 1).

**Table 1:** Baseline demographic, clinical, and treatment characteristics and functional outcomes at hospital discharge.

	Variables	No. (%)
**Sex**	Male	18 (23%)
	Female	61 (77%)
**Age**	Mean ± SD	52 ± 11
**WFNS grade**	4	39 (49.4%)
	5	40 (50.6%)
**Aneurysm location**	ACOM	25 (32%)
	MCA	19 (24%)
	PCOM	10 (12%)
	PICA	7 (9%)
	ICA	7 (9%)
	Other	11 (14%)
**Treatment**	Clipping	51 (65%)
	Coiling	25 (31%)
	Other	3 (4%)
**mFisher**	2	4 (5%)
	3	22 (28%)
	4	53 (67%)
**GOS at discharge**	2	9 (11%)
	3	43 (54%)
	4	17 (22%)
	5	10 (12%)
**mRS at discharge**	0	3 (4%)
	1	8 (10%)
	2	15 (19%)
	3	5 (6%)
	4	39 (49%)
	5	9 (11%)
	6	0 (0%)

### 3.2 Long-term patient-reported outcomes

Compared with the 79 participating patients, the 44 eligible non-participating survivors did not differ significantly in age, sex, WFNS grade, modified Fisher grade, or treatment modality. However, functional outcome at hospital discharge differed significantly between the groups (p < 0.001), with 49% of non-participating patients having an mRS score of ≥5 compared with 11% of participating patients.

The mean SS-QOL score was 198.4 ± 42.4, the mean SF-12 PCS was 38.3 ± 6.96, and the mean PHQ-9 score was 5.4 ± 5.4. Nine patients (11.4%) had PHQ-9 scores ≥10, indicating at least moderate depressive symptomatology. An additional three patients had a documented history of depression and were receiving pharmacological or psychotherapeutic treatment despite PHQ-9 scores <10 at the time of assessment. Median follow-up was 4.36 years (IQR 2.51 – 8.04 years).

Using the predefined SS-QOL threshold, 9 out of the 79 included patients (11.4%) had SS-QOL scores ≤147, whereas 70 (88.6%) patients had SS-QOL scores >147. Descriptive comparison of SS-QOL domain scores between the two groups showed the largest absolute differences in mobility, self-care, upper-extremity function, social roles, and work/productivity (Fig. 2).

**Fig. 2:**
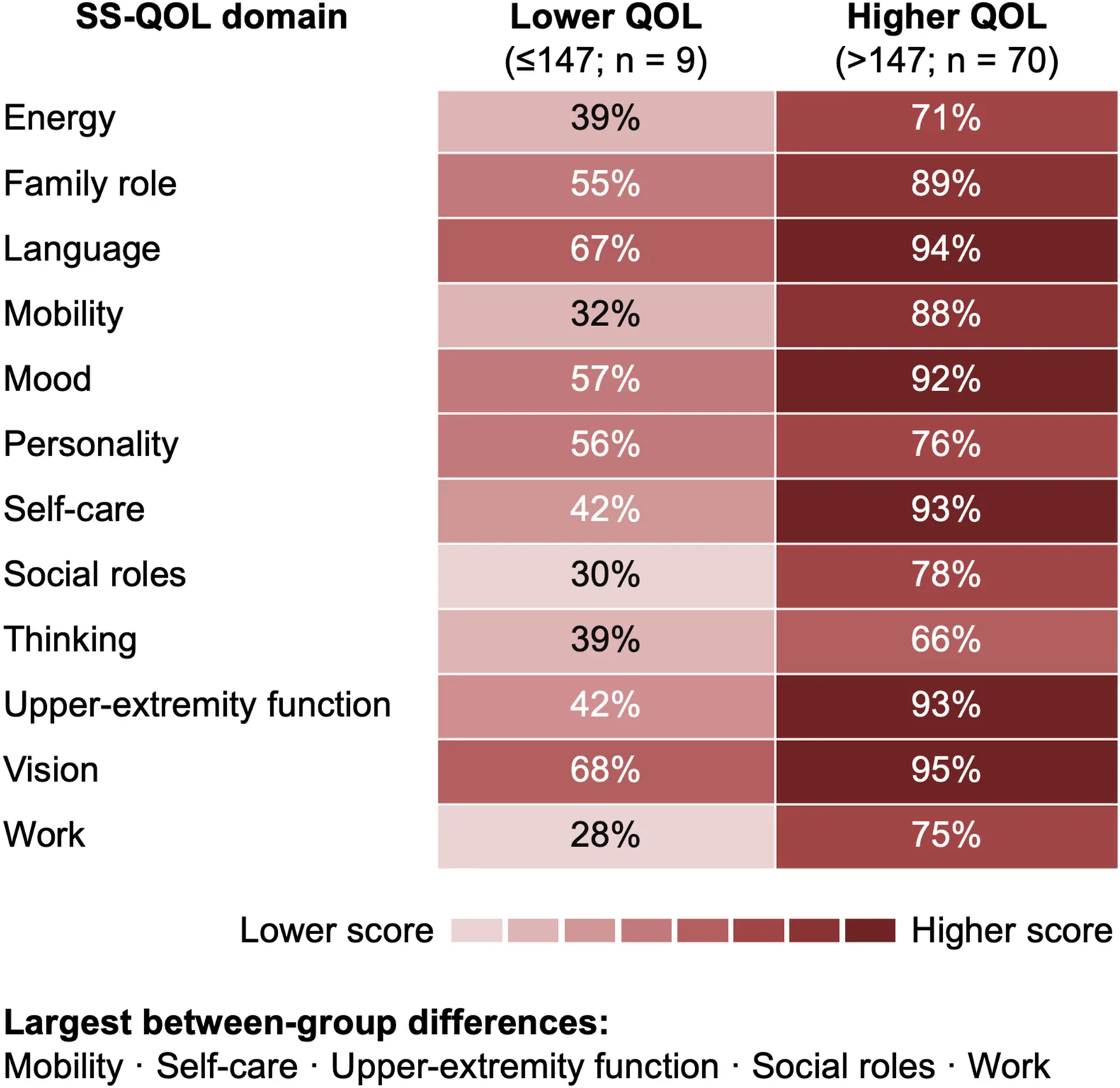
Descriptive comparison of SS-QOL domain scores between patients with lower and higher overall SS-QOL. Mean scores for the 12 SS-QOL domains are expressed as percentages of the maximum attainable score for each domain. Scores are shown for patients with SS-QOL ≤147 (n = 9) and SS-QOL >147 (n = 70). The figure illustrates descriptive differences in domain scores between the two predefined groups; no inferential statistical comparison was performed. Lighter and darker shades indicate lower and higher scores, respectively.

### 3.3 Factors associated with quality of life

In multiple linear regression with age, sex, WFNS score, mRS score at discharge, length of follow-up, history of depression, and total PHQ-9 score, total PHQ-9 score was the only variable significantly associated with SS-QOL (p < 0.001). Associations between PHQ-9 scores and individual SS-QOL domains were significant across all assessed domains, with the strongest Spearman correlations observed for family role (ρ = −0.62), work/productivity (ρ = −0.58), mood (ρ = −0.56), energy (ρ = −0.54), mobility (ρ = −0.52), and thinking (ρ = −0.51) (Table 2). Regression coefficients should be interpreted with caution because the assumptions underlying linear regression were not fully satisfied. SS-QOL and SF-12 PCS were positively correlated (ρ = 0.49, 95% CI 0.31 to 0.65, p < 0.001), whereas SS-QOL and PHQ-9 were negatively correlated (ρ = −0.67, 95% CI -0.78 to −0.53, p < 0.001) (Fig. 3). The SF-12 Mental Component Summary (MCS) was not significantly associated with overall SS-QOL (p = 0.41). The mean MCS score was 47.4 ± 7.2, which was close to the population norm.

**Table 2:** **Associations between depressive symptoms and SS-QOL domain scores. β:** linear regression coefficient for the normalized SS-QOL domain score; **CI:** confidence interval; **ρ:** Spearman's rank correlation coefficient; **p (raw):** uncorrected p-value for ρ; **p (corrected):** p-value for ρ after Benjamini–Hochberg correction for multiple testing. Because the assumptions for linear regression, including normality of residuals and homoscedasticity, were not fully satisfied, regression coefficients should be interpreted with caution.

Component (normalized to 0-1)	β (95% CI)	ρ (95% CI)	p (raw)	p (corrected)
Mood	−18.6 (−23.9 to −13.2)	−0.56 (−0.69 to −0.38)	<0.001	<0.001
Language	−16.4 (−23.5 to −9.4)	−0.50 (−0.65 to −0.31)	<0.001	<0.001
Family role	−15.8 (−20.4 to −11.2)	−0.62 (−0.74 to −0.46)	<0.001	<0.001
Vision	−15.2 (−21.6 to −8.8)	−0.46 (−0.62 to −0.27)	<0.001	<0.001
Self-care	−10.8 (−15.6 to −6.1)	−0.48 (−0.64 to −0.29)	<0.001	<0.001
Mobility	−10.5 (−14.8 to −6.3)	−0.52 (−0.66 to −0.33)	<0.001	<0.001
Work	−9.4 (−12.8 to −5.9)	−0.58 (−0.71 to −0.41)	<0.001	<0.001
Upper extremity function	−8.9 (−13.5 to −4.3)	−0.45 (−0.61 to −0.26)	<0.001	<0.001
Social roles	−8.8 (−12.7 to −4.9)	−0.48 (−0.63 to −0.29)	<0.001	<0.001
Thinking	−8.3 (−12.3 to −4.4)	−0.51 (−0.65 to −0.32)	<0.001	<0.001
Energy	−8.1 (−11.7 to −4.4)	−0.54 (−0.68 to −0.36)	<0.001	<0.001
Personality	−7.0 (−12.0 to −2.1)	−0.44 (−0.60 to −0.24)	<0.001	<0.001

**Fig. 3:**
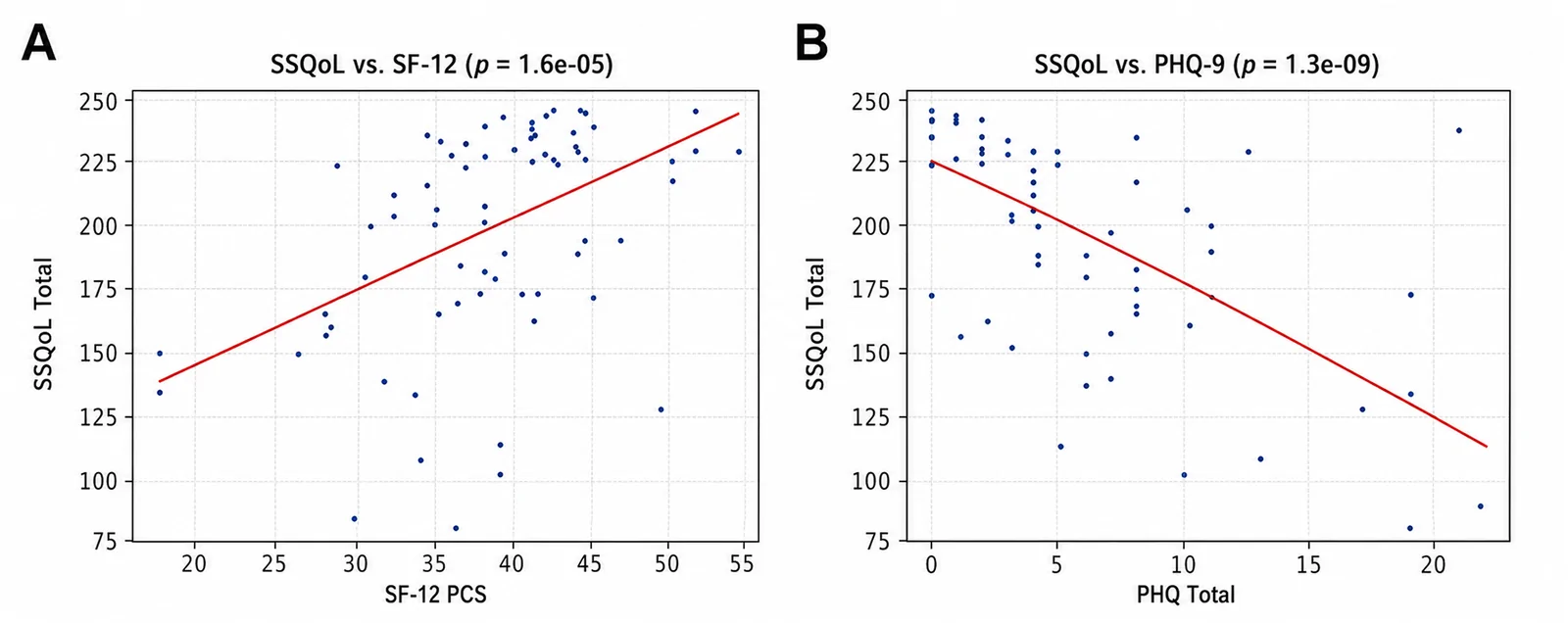
Correlations between SS-QOL and physical health and depressive symptoms. Scatter plots show the relationship between SS-QOL and (A) SF-12 Physical Component Summary (PCS) and (B) Patient Health Questionnaire-9 (PHQ-9) scores. Solid lines represent linear regression fits.

## 4 Discussion

Among the 339 patients presenting with poor-grade aneurysmal subarachnoid hemorrhage (aSAH), overall mortality was 47.8%. Long-term quality of life outcomes were assessed only in the subset of 79 survivors who were alive, eligible, and able to participate in follow-up. Among these selected long-term survivors, 88.6% had an SS-QOL score >147, indicating favorable quality of life within this group. These findings suggest that meaningful long-term recovery is achievable among participating survivors, despite an initially poor clinical presentation. However, this proportion does not reflect the overall poor-grade cohort. Patient-reported outcomes were necessarily restricted to survivors who were alive, eligible, and willing to participate. Moreover, non-participants included 25 patients with severe disability requiring institutional care, who may represent individuals with poorer long-term outcomes. Therefore, the observed quality-of-life outcomes should be interpreted as representing a selected cohort of long-term survivors rather than the entire population of patients with poor-grade aSAH.

### 4.1 Functional recovery

In this cohort of selected long-term survivors of poor-grade aSAH, differences in long-term quality of life were most pronounced in domains related to physical independence and participation in everyday activities. In the descriptive comparison between patients with SS-QOL ≤147 and > 147, the largest absolute differences were observed in mobility, self-care, upper-extremity function, social roles, and work/productivity. These findings suggest that physical independence and the ability to resume everyday and social roles are important dimensions of perceived recovery after poor-grade aSAH.

These findings are consistent with previous reports. Schwartz et al. demonstrated that poor-grade aSAH is not invariably associated with poor long-term outcome, reporting that approximately 60% of long-term survivors regained functional independence and perceived only moderately reduced quality of life compared with a healthy reference population (Schwartz et al., 2017). Likewise, in the 10-year follow-up of the ISAT cohort, functional dependency was the strongest predictor of impaired health-related quality of life, while Denneman et al. similarly demonstrated lower quality-of-life scores among poor-grade survivors with persistent disability (Hua et al., 2021; Denneman et al., 2025). Together, these observations reinforce the importance of maximizing functional recovery through timely neurocritical care, rehabilitation, and long-term support. These findings are particularly relevant when counseling families during the acute phase, as poor initial neurological grade does not necessarily preclude meaningful long-term recovery among survivors of the acute phase.

### 4.2 Cognitive and psychosocial domains

Long-term recovery after poor-grade aSAH appears inherently multidimensional, extending beyond physical independence to cognitive, emotional, and social domains. These aspects of recovery may not be fully captured by conventional functional outcome measures.

Our findings are broadly consistent with previous studies emphasizing the multidimensional nature of recovery after aSAH. Rass et al. reported persistent cognitive impairment in a substantial proportion of aSAH survivors and demonstrated an association between cognitive impairment and reduced quality of life (Rass et al., 2024). Although our study did not include formal neuropsychological testing and therefore cannot establish objective cognitive impairment, the significant associations between depressive symptoms and the SS-QOL thinking and language domains suggest that patient-perceived cognitive functioning forms an important component of long-term recovery. Similar observations have been reported by Bergamini et al., who demonstrated considerable variability in quality of life among SAH survivors despite comparable functional outcomes, emphasizing the value of patient-reported outcome measures alongside conventional functional assessments (Bergamini et al., 2025). Likewise, Persson et al. showed that many long-term SAH survivors experience persistent fatigue, cognitive, and emotional difficulties despite relatively preserved physical function and participation, underscoring the presence of hidden disabilities that may not be captured by traditional neurological outcome measures (Persson et al., 2025).

### 4.3 Depressive symptoms

Depressive symptoms were strongly associated with reduced long-term quality of life in our cohort, emphasizing the importance of psychological well-being in recovery after poor-grade aSAH. In the multivariable analysis, depressive symptom burden was the only variable significantly associated with overall SS-QOL, and higher PHQ-9 scores were inversely associated with all assessed SS-QOL domains, including physical, cognitive, emotional, and social dimensions. These findings suggest that the association between depressive symptoms and quality of life extends beyond emotional well-being to encompass patients’ perceptions of their physical functioning, cognitive abilities, and social participation years after the initial hemorrhage.

These findings are consistent with previous studies demonstrating a high prevalence of depressive symptoms after aSAH and their association with impaired quality of life (Visser-Meily et al., 2009). Rass et al. reported clinically relevant depressive symptoms in 16% of survivors, while Al-Khindi et al. identified depression and anxiety as predictors of poor functional outcome and reduced QOL (Rass et al., 2024; Al-Khindi et al., 2010a). Direct comparisons between studies should, however, be interpreted with caution because heterogeneity in assessment instruments (e.g., EQ-5D-3L, SF-36, SS-QOL, HADS, and PHQ-9) limit comparability. Interestingly, the SF-12 MCS was not significantly associated with overall SS-QOL, and the mean MCS score was close to the population norm. This finding should be interpreted in the context of the different constructs assessed by the two instruments: the SF-12 MCS provides a broad measure of perceived mental health-related quality of life, whereas the PHQ-9 specifically assesses depressive symptom severity. Thus, the absence of a significant association between MCS and SS-QOL does not necessarily contradict the strong association observed between PHQ-9 scores and SS-QOL. Depressive symptoms may represent a potentially modifiable aspect of long-term recovery. Although causality cannot be inferred from our cross-sectional analysis, our findings support consideration of routine psychological screening and appropriate mental health support during long-term follow-up in poor-grade aSAH.

## 5 Limitations

This study has several limitations. First, its retrospective single-center design as well as the relatively small cohort of 79 patients may limit generalizability. Second, the analysis is subject to substantial selection bias. Nearly half of the initial cohort died, and additional patients were either lost to follow-up or declined participation, particularly those with severe disability requiring institutional care. Importantly, non-participating survivors had significantly worse functional outcomes at hospital discharge than participating patients, with 49% having an mRS score of ≥5 compared with 11% among participants. This finding provides empirical evidence of selection bias and suggests that patients with greater functional impairment were underrepresented in the long-term patient-reported outcome assessment, potentially shifting the observed QOL toward more favorable values. As a result, the study population represents a selected subgroup of survivors with comparatively favorable outcomes. Third, the cohort was predominantly female (77%), reflecting the epidemiology of aneurysmal SAH but potentially limiting the generalizability of the findings to male patients. Also, QOL was assessed at a single time point, precluding evaluation of changes over time. Further, the study relied on patient-reported measures without formal neuropsychological testing. The SS-QOL cutoff value of 147 was validated in general stroke but not specifically in poor-grade aSAH. These limitations should be considered when interpreting the results, which primarily reflect outcomes among selected long-term survivors rather than the entire population of patients with poor-grade aSAH.

Finally, the exclusive use of quantitative, questionnaire-based assessments limits the depth of insight into patients’ lived experiences, particularly among patients with severe cognitive or physical impairments who cannot participate in survey-based follow-up. Qualitative or mixed-method approaches could provide additional insight into underrepresented dimensions of quality of life and may facilitate the inclusion of patients who are unable to participate in conventional questionnaire-based assessments.

## 6 Conclusion

In this cohort of selected long-term survivors of poor-grade aSAH, patient-reported quality of life was frequently favorable despite the severity of the initial hemorrhage. Depressive symptoms showed a strong association with lower long-term quality of life and were inversely associated with all assessed SS-QOL domains. Although substantial survivorship and selection bias limit the generalizability of these findings, they highlight that recovery after poor-grade aSAH is multidimensional and that comprehensive long-term follow-up should extend beyond functional assessment to include psychological well-being and patient-reported outcomes.

## Ethics declaration

Written informed consent to take part in the study and to publish the article has been obtained from all participants or their legal representatives. The privacy rights of participants have been observed.

This study was performed in compliance with relevant laws, regulatory frameworks and guidelines where the research took place.This study was approved by the Ethics Committee of the medical faculty at Heinrich-Heine University Düsseldorf.(Approval No. 2024–2822)

## Declaration of generative AI and AI-assisted technologies in the manuscript preparation process

During the preparation of this work, the authors used ChatGPT (OpenAI) for assistance with language editing and manuscript organization. The authors reviewed and edited the output as needed and take full responsibility for the content of the published article.

## Funding

None.

## Declaration of competing interests

The authors declare that they have no known competing financial interests or personal relationships that could have appeared to influence the work reported in this paper.

## Abbreviations

**aSAH**, aneurysmal subarachnoid hemorrhage; **AI**, artificial intelligence; **CI**, confidence interval; **CT**, computed tomography; **CTA**, computed tomography angiography; **CTP**, computed tomography perfusion; **CVS**, cerebral vasospasm; **DCI**, delayed cerebral ischemia; **DSM-5**, Diagnostic and Statistical Manual of Mental Disorders, Fifth Edition; **EQ-5D-3L**, EuroQol 5-Dimension 3-Level questionnaire; **FDR**, false discovery rate; **GCS**, Glasgow Coma Scale; **GOS**, Glasgow Outcome Scale; **HADS**, Hospital Anxiety and Depression Scale; **ICA**, internal carotid artery; **IQR**, interquartile range; **ISAT**, International Subarachnoid Aneurysm Trial; **IV**, intravenous; **MCA**, middle cerebral artery; **MCS**, Mental Component Summary; **mFisher**, modified Fisher grade; **mRS**, modified Rankin Scale; **PCOM**, posterior communicating artery; **PCS**, Physical Component Summary; **PHQ-9**, Patient Health Questionnaire-9; **PICA**, posterior inferior cerebellar artery; **QOL**, quality of life; **SAH**, subarachnoid hemorrhage; **SD**, standard deviation; **SF-12**, 12-Item Short Form Health Survey; **SF-36**, 36-Item Short Form Health Survey; **SS-QOL**, Stroke-Specific Quality of Life; **WFNS**, World Federation of Neurosurgical Societies.
